# Significance of thyroid dysfunction in the patients with primary membranous nephropathy

**DOI:** 10.1186/s12882-022-03023-y

**Published:** 2022-12-10

**Authors:** Qiu-hua Gu, Xin Cao, Xiao-ming Mao, Jun-ya Jia, Tie-kun Yan

**Affiliations:** 1 Department of Nephrology, Tianjin Medical University General Hospital, Tianjin, 300052 China; 2 Department of Nephrology, Tianjin Medical University General Hospital Airport Hospital, Tianjin, 300308 China

**Keywords:** Membranous nephropathy, Thyroid dysfunction, Anti-PLA2R antibody

## Abstract

**Background:**

Thyroid dysfunction is common in patients with nephrotic syndrome, especially patients with primary membranous nephropathy (pMN). In view of both MN and thyroid dysfunction are associated with autoimmunity, the current study aimed to elucidate the significance of thyroid dysfunction in patients with pMN.

**Methods:**

Four hundred and twenty patients with biopsy-proven pMN from 2018–2021 were retrospectively enrolled. Clinical and pathological parameters, and treatment response of patients with and without thyroid dysfunction were analyzed.

**Results:**

Ninety-one (21.7%) patients with pMN suffered from thyroid dysfunction, among which subclinical hypothyroidism (52.7%) was the main disorder. Compared to patients with normal thyroid function, patients with thyroid dysfunction presented with a higher level of proteinuria, a lower level of serum albumin, a higher level of serum creatinine and more severe tubulointerstitial injury at the time of biopsy. But the positive rate and level of circulating anti-phospholipase A2 receptor (PLA2R) antibody were comparable between these two groups. Though following the similar treatment, the percentage of no response to treatment were significantly higher in the patients with thyroid dysfunction (38.6 vs. 20.0%, *P* = 0.003). Similar to the urinary protein and the positivity of anti-PLA2R antibody, multivariate COX analysis showed thyroid dysfunction was also identified as an independent risk factor for the failure to remission (HR = 1.91, 95%CI, 1.07–3.40, *P* = 0.029).

**Conclusion:**

In conclusion, thyroid dysfunction is common in the patients with pMN and might predict a severe clinical manifestation and a poor clinical outcome, which indicated that the thyroid dysfunction might be involved in the disease progression of pMN.

## Introduction

It is commonly known that hypothyroidism usually occurs in patients with proteinuria, in particular nephrotic syndrome. In addition to a series of cases [1, 2], several cohort studies also reported that patients with massive proteinuria may develop subclinical hypothyroidism or overt hypothyroidism [3–7]. Li et al. found the incidence of thyroid dysfunction, including subclinical hypothyroidism, overt hypothyroidism and low T3/T4 syndrome in patients with nephrotic syndrome could be as high as 82.0% [4]. Notably, the main pathological type of patients with nephrotic syndrome and thyroid dysfunction was membranous nephropathy [4, 5], which is the main cause of nephrotic syndrome in adult and characterized by the accumulation of glomerular subepithelial immune complexes deposition and the basement membrane thickening [8].

The main cause of thyroid dysfunction in patient with proteinuria was usually thought as the urinary loss of thyroid hormone [2], for its link to serum albumin. Reversely, it is reported that 10–30% patients with autoimmune thyroid disease could also involve with glomerular injury [9]. And the most common associated glomerular disease was membranous nephropathy, too [10]. Considering that both membranous nephropathy and thyroid dysfunction could be caused by autoimmune disorder, whether there was any special significance of thyroid dysfunction in patients with pMN remains unclear. In the current study, we investigated the clinical and pathological features of patients with combined pMN with thyroid dysfunction in a large consecutive cohort. Our findings are beneficial for understanding the significance of thyroid dysfunction in patients with pMN.

## Methods

### Patients

**Fig. 1:**
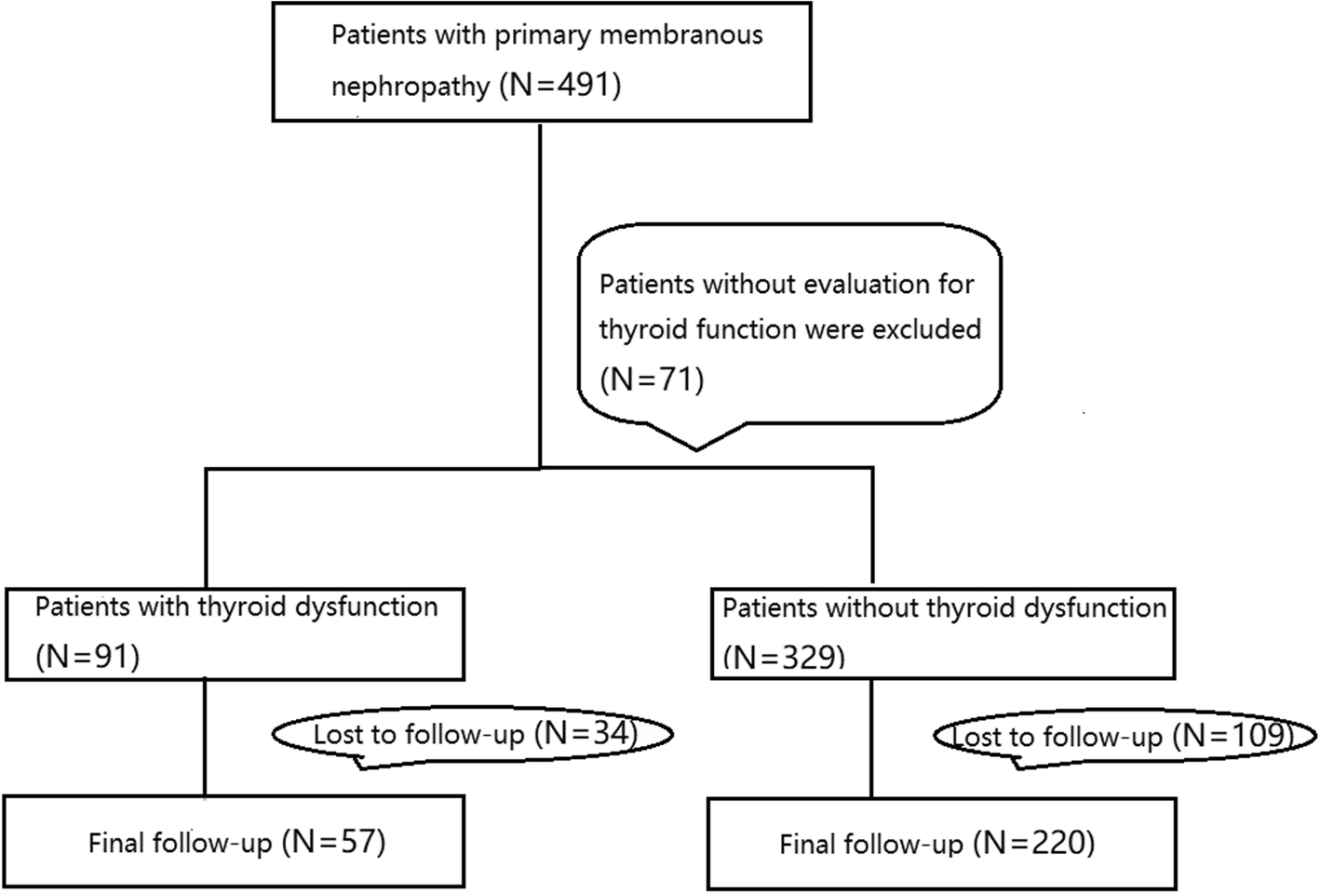
Flow chart of the enrollment

Four hundred and ninety-one consecutive patients with biopsy-proven pMN, diagnosed in Tianjin Medical University General Hospital from 2018 to 2021 were reviewed retrospectively. Patients with membranous nephropathy secondary to known factors, such as malignant tumors, hepatitis virus B/C, systemic lupus erythematosus or heavy metal poisoning, were carefully excluded. Patients with known autoimmune thyroid disease, hypothalamic-pituitary axis dysfunction, pituitary tumors, absent thyroid gland, thyroid gland tumors before the onset of membranous nephropathy were also excluded from the study. Among them, thyroid function of 71 patients were not evaluated at the time of biopsy. Thus, 420 patients remained in this study (Fig. 1). The latest clinical data, including the free triiodothyronine (FT3), free thyroxine (FT4) and thyroid-stimulating hormone (TSH), before renal biopsy and during follow-up were collected from the medical records. Estimated glomerular filtration rate (eGFR) was calculated from serum creatinine levels using the Chronic Kidney Disease Epidemiology Collaboration (CKD-EPI) equation [11]. In this study, thyroid dysfunction consisted of hypothyroidism, subclinical hypothyroidism, and low T3 and/or T4 syndrome. Hypothyroidism was defined as a higher level of TSH combined with a lower level of FT4; subclinical hypothyroidism was defined as a higher level of TSH combined with a normal level of FT4; while low T3 and/or T4 syndrome was defined as only a merely lower FT3 and/or FT4. The research was in compliance of the Declaration of Helsinki and approved by the ethics committee of our hospital.

### Renal histopathology

Renal biopsy was performed for all the patients at the time of diagnosis. Renal specimens were evaluated by direct immunofluorescence, light microscopy, and electron microscopy.

For direct immunofluorescence, IgG, IgM, IgA, C3c, C1q and fibrinogen were detected by fluorescein isothiocyanate (FITC)-conjugated antibodies (Dako, Copenhagen, Denmark) on frozen tissues. PLA2R expression in glomeruli was detected by indirect immunofluorescence with rabbit polyclonal anti-PLA2R antibodies as the primary antibody (Atlas Antibodies, Stockholm, Sweden) on frozen tissues.

For light microscopy, paraffin sections were stained with hematoxylin and eosin, periodic acid-Schiff, periodic acid-silver methenamine, and Masson’s trichrome. Pathologic findings in the glomeruli, tubules, interstitium, and vessels were described in detail. Stages of MN were defined according to the Churg and Ehrenreich’s classification criteria. The chronic tubulointerstitial injury was defined as tubular atrophy and interstitial fibrosis. The scoring of chronic tubulointerstitial injury were graded semi-quantitatively from 0 to 2: 0, normal; 1, < 25.0% of interstitia affected; 2, > 25.0% of interstitia affected.

### Detection of anti-PLA2R antibody

Circulating anti-PLA2R antibody was detected in the automatic analyzer, using a commercial quantum-dots based fluorescence-linked immunosorbent assay kit (Nanjing vazyme medical technology co.ltd, Nanjing, China) with the recombinant human PLA2R as the antigen, following the standard instructions recommended by the manufacturers. The level of anti-PLA2R antibody > 14RU/ml was designated as positivity.

### Treatments and outcomes

Patients with pMN were treated according to the Kidney Disease: Improving Global Outcomes (KDIGO) guidelines for membranous nephropathy. During follow-up, for evaluation of therapeutic responses, complete remission was defined as urinary protein excretion < 0.3 g/24 h with normal renal function. Partial remission was defined as urinary protein excretion < 3.5 g/24 h but > 0.3 g/24 h, or at least a 50% reduction from peak values with stable serum creatinine (no more than 25% increase from baseline). Treatment failure was defined as not reaching the criteria of partial remission. For evaluation of treatment outcomes, treatment failure was used as the primary endpoint. End-stage kidney disease(ESKD) (defined as eGFR < 15 ml/min*1.73m^2^ or dependence on dialysis) and eGFR decreasing more than 25% from baseline were used to evaluate the renal function.

### Statistical analysis

All the statistical analyses were performed using SPSS statistical software package, version 13.0 (SPSS Inc., Chicago, IL, USA). Differences of quantitative parameters were assessed using the t-test for data that were normally distributed or nonparametric test for data that were not normally distributed. Differences of qualitative data were compared using the chi-square test/Fisher’s exact test, otherwise, the Kruskal–Wallis or Mann–Whitney U tests were performed. Correlations of quantitative parameters were analyzed using the Pearson correlation coefficient analysis for data that were normally distributed and Spearman correlation coefficient analysis for data that were not normally distributed. Kaplan–Meier curves were used to analyze the clinical treatment outcomes of patients (Log-rank test). If the *P* value of the candidate predictor in univariate survival analysis was less than 0.05, this predictor would be included into the multivariable Cox regression models. All statistical analyses were two-tailed and *P* value < 0.05 was considered significant.

## Results

### Prevalence and features of thyroid dysfunction in patients with pMN

Four hundred and twenty consecutive patients with renal biopsy-proven pMN were enrolled in this study. Among them, 91 (21.7%) patients suffered from thyroid dysfunction, including 48 (52.7%) patients with subclinical hypothyroidism, 16 (17.6%) patients with overt hypothyroidism, and 27 (29.7%) patients with low T3 and/or low T4 syndrome. The other 329 (78.3%) patients presented with normal thyroid function. Compared to the patients with normal thyroid function, patients with thyroid dysfunction had significantly lower levels of FT3 (3.3 ± 0.9 vs. 3.8 ± 0.6 pmol/L, *P* < 0.001) and FT4 (9.9 ± 1.8 vs. 11.1 ± 1.3 pmol/L, *P* < 0.001), but a higher level of TSH (5.7, 3.6–8.2 vs. 2.5, 1.7–3.2 uIU/ml, *P* < 0.001) (Table1).

**Table 1:** The demographic and clinical characteristics of pMN patients with and without thyroid dysfunction

	Patients with thyroid dysfunction (n = 91) (21.7%)	Patients with normal thyroid function (n = 329)( 78.3%)	*P*
Male/female	56/35	191/138	0.550
Age (years)	53.3 ± 11.6	50.8 ± 13.6	0.092
Time from onset to biopsy(months)	3.0 (1.0–6.0)	4.0 (1.0–12.0)	0.199
Hypertension, n (%)	45 (49.5)	160 (48.6)	0.890
Urinary protein (g/24 h)	6.0 (2.5–9.6)	4.4 (2.2–6.6)	**0.001**
Serum albumin (g/L)	21.3 ± 6.9	25.1 ± 6.6	** < 0.001**
Serum cholesterol (mmol/L)	8.1 (6.4–10.2)	6.8 (5.6–8.4)	** < 0.001**
Serum triglyceride (mmol/L)	2.4 (1.7–3.2)	2.2 (1.5–3.2)	**0.051**
Nephrotic syndrome, n (%)	61 (67.0)	170 (51.7)	**0.009**
Hemoglobin (g/L)	128.6 ± 21.2	135.6 ± 18.4	**0.015**
Hematuria, n (%)	54/80 (67.5)	201/305 (65.9)	0.331
Serum creatinine (umol/L)	78.5 ± 40.1	66.0 ± 19.8	**0.003**
eGFR (ml/min*1.73m^2^)	92.2 ± 23.5	102.7 ± 17.8	** < 0.001**
Free Triiodothyronine (pmol/L)	3.3 ± 0.9	3.8 ± 0.6	** < 0.001**
Free thyroxine (pmol/L)	9.9 ± 1.8	11.1 ± 1.3	** < 0.001**
Thyroid-stimulating hormone (uIU/ml)	5.7 (3.6–8.2)	2.5 (1.7–3.2)	** < 0.001**
Serum C3 (mg/L)	91.7 ± 21.8	94.9 ± 17.7	0.761
Serum C4 (mg/L)	26.4 ± 7.4	25.7 ± 7.6	0.233
Serum IgG (mg/L)	560.0 (443.8–828.3)	656.5 (478.8–832.0)	0.077
Positivity of anti-PLA2R antibody, n (%)	53/75 (58.2)	175/272 (53.2)	0.593
Serum anti-PLA2R antibody, (RU/ml)	47.6 (8.6–174.7)	37.7 (5.8–104.8)	0.178

MN: membranous nephropathy; PLA2R: phospholipase A2 receptor

### Clinical characteristics of pMN patients combined with thyroid dysfunction

The demographic and clinical data of patients with and without thyroid dysfunction were shown in Table 1. Among the 91 patients with thyroid dysfunction, 56 patients were male and 35 were female, with a mean age of 53.3 ± 11.6 years, comparable to that of patients with normal thyroid function (53.3 ± 11.6 vs. 50.8 ± 13.6 years, *P* = 0.092). Compared to the patients with normal thyroid function, we found patients combined with thyroid dysfunction had a significantly higher percentage of nephrotic syndrome (67.0% vs. 51.7%, *P* = 0.009), presenting with more proteinuria (6.0, 2.5–9.6 vs. 4.4, 2.2–6.6 g/24 h, *P* = 0.001), a higher level of serum cholesterol (8.1, 6.4–10.2 vs. 6.8, 5.6–8.4 mmol/L, *P* < 0.001), while a lower level of serum albumin (21.3 ± 6.9 vs. 25.1 ± 6.6 g/L, *P* < 0.001). In addition, the kidney function was poorer in pMN patients combined with thyroid dysfunction, with a significantly higher level of serum creatinine (78.5 ± 40.1 vs. 66.0 ± 19.8 umol/L, *P* = 0.003) and lower level of eGFR (92.2 ± 23.5 vs. 102.7 ± 17.8 ml/min*1.73m^2^, *P* < 0.001). The level of hemoglobin of patients combined with thyroid dysfunction was 128.6 ± 21.2 g/L, significantly lower than that of patients with normal thyroid function (128.6 ± 21.2 vs. 135.6 ± 18.4 g/L, *P* = 0.015). Among all the patients, circulating anti-PLA2R antibody was detected in 347 patients, including 75 patients with thyroid dysfunction and 272 patients with normal thyroid function. The positive rate of circulating anti-PLA2R antibody at the time of diagnosis was similar in pMN patients with and without thyroid dysfunction (58.2 vs. 53.2%, *P* = 0.593). There was no significant difference in the level of anti-PLA2R antibody between these two groups, either (47.6, 8.6–174.7, 37.7, 5.8–104.8 RU/ml, *P* = 0.178) (Table 1).

The time from onset to biopsy, the prevalence of hypertension and hematuria, the level of serum triglyceride, serum IgG, serum C3 and serum C4 were comparable between these two groups (Table 1).

**Fig. 2:**
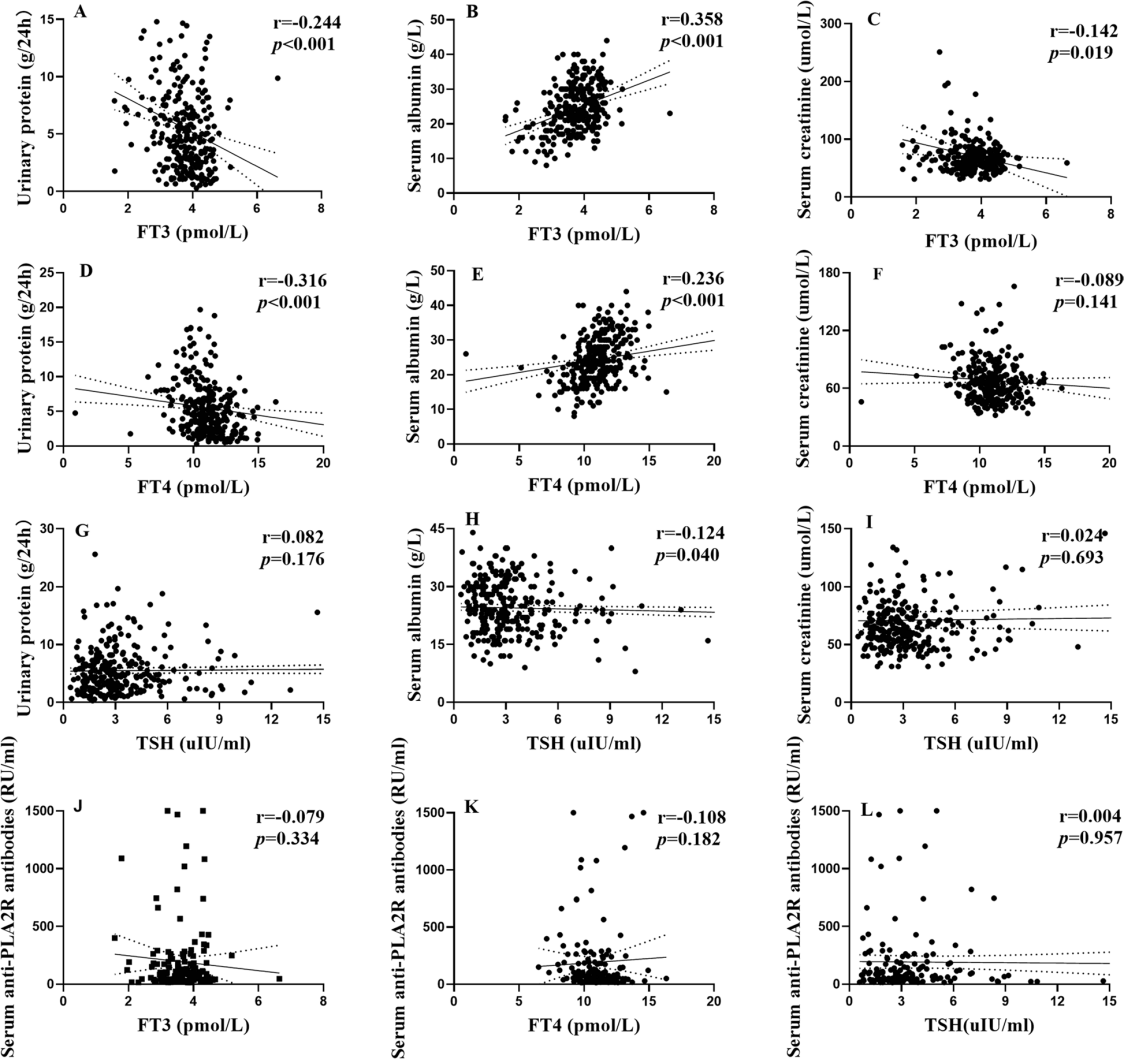
Correlations between the levels of FT3, FT4, TSH and the clinical parameters of patients with pMN

We also analyzed the correlation between FT3, FT4, and TSH and the clinical parameters of patients with pMN. We found the level of FT3 correlated negatively with urinary protein (r = -0.244, *P* < 0.001) and correlated positively with serum albumin (r = 0.358, *P* < 0.001) (Fig. 2A-B). The level of FT3 also associated negatively with serum creatinine (r = -0.142, *P* = 0.019) (Fig. 2C). The level of FT4 also showed a similar association with urinary protein (r = -0.316, *P* < 0.001) and serum albumin (r = 0.236, *P* < 0.001), but did not related to serum creatinine (r = -0.089, *P* = 0.141) (Fig. 2D-F). However, we found the level of TSH only correlated negatively with serum albumin (r = -0.124, *P* = 0.040), while it had no significant correlation with urinary protein (r = 0.082, *P* = 0.176) and the parameters of renal function (r = 0.024, *P* = 0.693) (Fig. 2G-I). But the levels of FT3, FT4, TSH had no relationship with circulating anti-PLA2R antibody level (Fig. 2J-L).

For the retrospective study, only 17/91 patients with thyroid dysfunction had anti- thyroglobulin antibody (TGAb) and anti-thyroid peroxidase antibody (TPOAb) detection record. Five of seventeen (29.4%) patients were TGAb positive and 6/17 (35.3%) patients were TPOAb positive. In the 17 patients, anti-PLA2R antibody was detected in 13 patients, and 3/9 (33.3%) patients with anti-PLA2R antibody positivity were TGAb/TPOAb positive, while in the 4 anti-PLA2R antibody negative patients, only 1 (25.0%) patient was TGAb/TPOAb positive. Nine of ninety-one patients had anti-thyrotropin receptor antibody (TRAb) detection record, but all of them were TRAb negative.

### Pathological features of pMN patients with thyroid dysfunction

All the patients were biopsy-proven pMN by light microscopy, immunofluorescence and electron microscopy. By immunofluorescence, we found there was no significant difference in glomerular IgG deposition, IgA deposition, IgM deposition, C3 deposition or C1q deposition between patients with and without thyroid dysfunction (Table 2). The glomerular PLA2R expression was detected in 55 patients by immunofluorescence. The positive rate of PLA2R staining was comparable between these two group (72.7 vs. 81.8%, *P* = 0.674). By light microscopy, the Churg stages of the 91 patients with thyroid dysfunction consisted of 29 (31.9%) patients of stage I, 33 (36.2%) patients of stage II and 29 (31.9%) patients of stage III, with no significant difference with that of patients with normal thyroid function (*P* = 0.629) (Table 2). The percentage of sclerosis lesions in glomeruli was also comparable between the patients with and without thyroid dysfunction (5.6, 0.0–14.3% vs. 5.9, 0.0–13.5%, *P* = 0.860) (Table 2). But it was worth noting that for the patients with thyroid dysfunction, 14/91 (15.4%) patients showed moderate chronic tubulointerstitial injury, which was more common than that of patients with normal thyroid function (15.4 vs. 5.2%, *P* = 0.004). (Table 2).

**Table 2:** The pathologic features of pMN patients with and without thyroid dysfunction

	Patients with thyroid dysfunction (n = 91)	Patients with normal thyroid function (n = 329)	*P*
MN stages, n (%)			0.629
I	29 (31.9)	118 (35.9)	
II	33 (36.2)	122 (37.1)	
III	29 (31.9)	89 (27.0)	
IgG deposition, n (%)	91 (100.0)	329 (100.0)	1.000
IgA deposition, n (%)	21 (23.1)	49 (14.9)	0.064
IgM deposition, n (%)	89 (97.8)	324 (98.5)	0.655
C3 deposition, n (%)	91(100.0)	329 (100.0)	1.000
C1q deposition, n (%)	28 (30.8)	96 (29.2)	0.769
Percentage of sclerosis, n (%)	5.6 (0.0–14.3)	5.9 (0.0–13.5)	0.860
PLA2R staining, n (%)	8/11 (72.7)	36/44 (81.8)	0.674
Chronic tubulointerstitial injury, n (%)			**0.004**
Grade 0	1 (1.1)	0 (0.0)	
Grade 1	76 (83.5)	312 (94.8)	
Grade 2	14 (15.4)	17 (5.2)	

### Treatment responses

During a mean follow-up of 17.6 ± 11.9 months, 34 (37.4%) patients with thyroid dysfunction and 109 (35.1%) patients with normal thyroid function were lost to follow-up. The mean follow-up time was similar between patients with and without thyroid dysfunction (17.6 ± 11.9 vs. 17.2 ± 10.3 months, *P* = 0.795). During follow-up, of the 57 patients with thyroid dysfunction, 48 (84.2%) patients received steroids and/or immunosuppressive drugs, which was similar to that of those with normal thyroid function (84.2% vs. 82.3%, *P* = 0.984) (Table 3).

**Table 3:** Treatment and outcomes of pMN patients with and without thyroid dysfunction

	Patients with thyroid dysfunction (n = 57) (62.6%)	Patients with normal thyroid function (n = 220) (64.9%)	*P*
Treatments			0.984
ACEI/ARBs only, n (%)	9 (15.8)	39 (17.7)	
Corticosteriods + cyclophosphamide	22 (38.6)	84 (38.2)	
Calcineurin inhibitor w/o corticosteroids	20 (35.1)	73 (33.2)	
Rituximab	6 (10.5)	24 (10.9)	
Response to treatment			0.013
Complete remission, n (%)	10 (17.5)	47 (21.4)	
Partial remission, n (%)	25 (43.9)	129 (58.6)	
No response, n (%)	22 (38.6)	44 (20.0)	
Decreased eGFR > 25%, n (%)	4 (7.0)	12 (5.5)	0.895
ESKD, n (%)	0 (0)	2 (9.0)	1.000
Survival time of normal renal function (months)	16.4 ± 11.6	16.2 ± 10.0	0.934
Follow up time (months)	17.6 ± 11.9	17.2 ± 10.3	0.795

ESKD: end-stage kidney disease

**Fig. 3:**
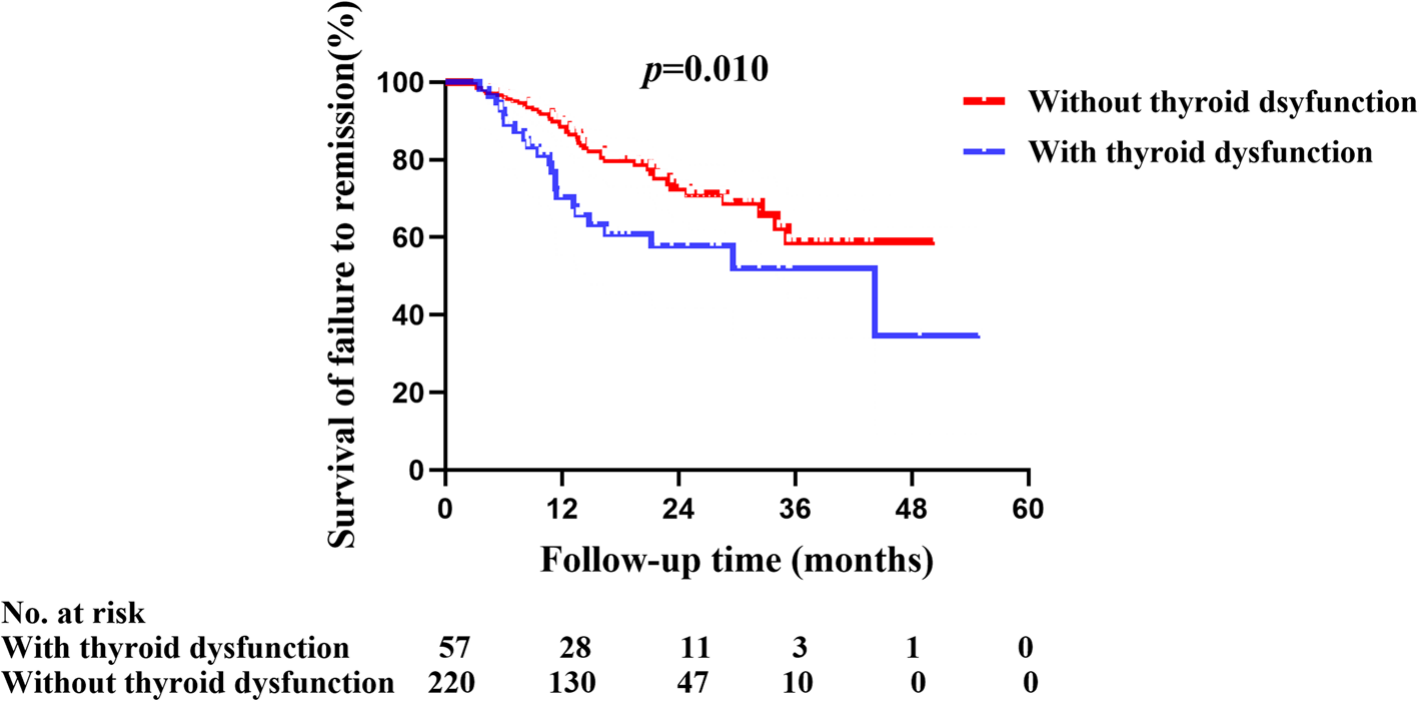
Kaplan–Meier analysis of failure to remission in pMN patients with and without thyroid dysfunction

After treatments, 35/57 (61.4%) patients with dysfunction obtained remission, including 10 (17.5%) patients with complete remission and 25 (43.9%) patients with partial remission. The rate of remission was significantly lower than that of patients with normal thyroid function (61.4 vs. 80.0%, *P* = 0.013) (Table 3). The percentage of no response to treatment in patients with thyroid dysfunction was significantly higher than that of patients with normal thyroid function (38.6 vs. 20.0%, *P* = 0.003). Kaplan–Meier curve also revealed that the cumulative survival of failure to treatment in the patients with thyroid dysfunction was significantly lower than that of those with normal thyroid function (*P* = 0.010) (Fig. 3).

Using univariate Cox regression analysis, thyroid dysfunction was identified as one of the risk factors for no remission of patients with pMN (HR = 1.84, 95%CI, 1.10–3.10, *P* = 0.021). Furtherly, multivariate analysis identified thyroid dysfunction as one of the independent risk factors for worse treatment response (HR = 1.91, 95%CI, 1.07–3.40, *P* = 0.029). The other independent risk factors included the positivity of anti-PLA2R antibody (HR = 2.95, 95%CI, 1.30–6.71, *P* = 0.010), the higher level of urinary protein (HR = 1.09, 95%CI, 1.02–1.17, *P* = 0.016). Compared with other pathological parameters, the severity of chronic tubulointerstitial injury on renal biopsy was also verified as a risk factor for clinical outcome of patients with pMN (HR = 2.44, 95%CI 1.27–4.69, *P* = 0.007). However, it was not the independent risk factor after multivariate analysis. (Table 4).

**Table 4:** Risk factors for no remission in patients with pMN

	Univariate analysis		Multivariate analysis	
	*P*-value	HR (95%CI)	*P*-value	HR (95%CI)
Female/male	**0.018**	1.89 (1.12–3.21)	0.210	1.54 (0.78–3.04)
Age (per1 year)	0.088	1.02 (1.00–1.03)	0.062	1.02 (1.00–1.04)
Anti-PLA2R antibody				
Negative	Reference			
Positive	**0.003**	3.36 (1.52–7.43)	**0.010**	2.95 (1.30–6.71)
Urinary protein (per 1 g/24 h)	** < 0.001**	1.12 (1.06–1.17)	**0.016**	1.09 (1.02–1.17)
Serum albumin (per 1 g/L)	**0.003**	0.95 (0.91–0.98)	0.777	0.99 (0.95–1.04)
Serum creatinine (per 1umol/L)	**0.001**	1.01 (1.00–1.02)	0.356	1.01 (1.00–1.02)
eGFR (per 1 ml/min*1.73m2)	** < 0.001**	0.98 (0.97–0.99)		
Thyroid function				
Normal thyroid function	Reference			
Thyroid dysfunction	**0.021**	1.84 (1.10–3.10)	**0.029**	1.91 (1.07–3.40)
Chronic tubulointerstitial injury (per 1 +)	**0.007**	2.44 (1.27–4.69)	0.789	1.15 (0.41–3.24)

### Renal outcomes

During the follow-up period, no patient with thyroid dysfunction progressed to ESKD, while 2 patients in the group with normal thyroid function received dialysis (0.0 vs. 9.0%, *P* = 1.000). Among the 57 patients with thyroid dysfunction, the eGFR of 4 (7.0%) patients showed a reduction of more than 25%, with no significant difference with that of patients with normal thyroid function (7.0 vs.5.5%, *P* = 0.895). (Table 3).

## Thyroid function outcome

During follow-up, 34/57 patients with thyroid dysfunction had the thyroid function re-evaluated. Among them, we found 10 patients received the thyroid hormone replacement, 7 of whom were patients with overt hypothyroidism. Compared to the patients with replacement, patients without replacement had a similar remission of both thyroid dysfunction (54.2 vs. 60.0%, *P* = 1.000) and proteinuria (66.7 vs. 70.0%, *P* = 1.000). Nineteen patients got normal thyroid function during follow-up, including 14/23 patients with proteinuria remission and 5/11 patients with consistent proteinuria (60.9 vs. 45.5%, *P* = 0.475).

## Discussions

Thyroid dysfunction is not uncommon in patients with nephrotic syndrome, and membranous nephropathy seems to be the most relevant primary glomerular disease in adult. But the clinical significance of thyroid dysfunction in patients with pMN in a larger cohort has never been well elucidated yet.

In this study, we found 91/420 (21.7%) patients with pMN suffered from thyroid dysfunction, and subclinical hypothyroidism 48/420 (11.4%) was the dominant disorder. These results were consistent with other studies in patients with proteinuria [3, 6, 7]. However, Li et found 260/317 (82.0%) patients with nephrotic syndrome showed abnormal thyroid function, consisting of 61 (19.2%) patients with subclinical hypothyroidism, 83 (26.2%) patients with overt hypothyroidism, and 116 patients (36.6%) with euthyroid sick syndrome [4]. The obvious higher prevalence of thyroid dysfunction in this study may be for that the patients in this study had a higher level of proteinuria, and a variety of the pathological types. All of that indicated that thyroid dysfunction was a commonly concomitant manifestation in patients with pMN.

Urine loss of thyroid hormones linked with plasma protein was thought as the original reason for the hypothyroid dysfunction in patients with proteinuria [12–16]. In the current study, we found patients with combined thyroid dysfunction had a higher level of urinary protein and lower level of serum albumin than that of patients with normal thyroid function. The levels of FT3 and FT4 correlated positively with the level of urinary protein and negatively with serum albumin. The level of TSH also associated with the serum albumin. Similar results were found in almost all the previous studies [1, 3, 4, 6, 7]. These findings seemed to indicate that the thyroid dysfunction in patients with pMN might be also secondary to the loss of thyroid hormones from urine, as thought in studies of patients with proteinuria [3, 14, 15, 17].

Previous studies found membranous nephropathy was also the most common pathological type in the autoimmune thyroiditis associated glomerulonephritis [10]. Both thyroglobulin (TG) and thyroperoxidase (TPO) had been found in the subepithelial immune deposits, as the main characteristic of membranous nephropathy, which may be the reason of the higher prevalence of membranous nephropathy associated with autoimmune thyroid disease [18–20]. Despite all the patients with known thyroid disease were excluded, we found 6/17 (35.3%) patients with thyroid dysfunction had TGAb and/or TPOAb. Jain et al. found 25/60 (41.7%) patients with nephrotic syndrome were TPOAb positive, who also suffered from severe proteinuria and poor renal function [5]. All of these indicated that the thyroid dysfunction of patients with pMN might not only be caused by urinary protein, but also involved with the coexistence of autoimmune thyroiditis. But whether there are some other mutual antigens linked MN with hypothyroidism is still unknown.

Megalin is a protein expressed on the apical surface of thyroid epithelial cells [20], which is also a possible immunologic target involved in the immunopathogenesis of glomerular injury in experimental Heymann model [21]. However, it is not the autoantigen of patients with membranous nephropathy, leading to a failure of connecting thyroid dysfunction with membranous nephropathy. Phospholipase A2 receptor (PLA2R) expressed in the podocyte has been identified as the main autoantigen of pMN [22]. Recently, by using a highly sensitive time-resolved fluoroimmunoassay, Huang et al. found the positive rate of anti-PLA2R antibody in the patients with thyroid disease and pMN was 97.50% and 82.61%, respectively [23]. Immunohistochemistry also revealed an obvious staining of PLA2R in tissues from patients with Hashimoto’s thyroiditis, with a positive rate of 66.67% [23]. These results indicated PLA2R as a potential pathogenic target antigen for both MN and autoimmune thyroid disease. However, in the current study, we found there was no significant difference of the positive rate or the level of anti-PLA2R antibody in the patients with or without thyroid dysfunction. Whether PLA2R is the underling mutual autoantigen between MN and autoimmune thyroid disease needs further investigation.

Based on previous studies and the findings in our study, thyroid dysfunction is common in MN. But whether thyroid dysfunction had a worse impact in the clinical outcomes of patients with pMN was also meaningful. In the current study, our results showed that patients with thyroid dysfunction had worse renal function, which might be the result of change in renal blood caused by the hypothyroidism [24]. Because thyroid hormones influence not only renal development, but also renal hemodynamics, GFR, and sodium and water homeostasis. A cross-sectional cohort of 74,356 adults by Chang et al. found subclinical hypothyroidism was a novel risk factor of reduced renal function [25]. We also found despite a strong association with the level of urinary protein, thyroid dysfunction was an independent risk factor for failure to remission. Thus, we speculated the subclinical or overt hypothyroidism may have a worse impact on the progression of MN in return.

Though our study also showed patients without thyroid hormone replacement had a similar remission of proteinuria to that of patient with replacement. A meta-analysis found thyroid hormone replacement therapy significantly increased the remission of nephrotic syndrome in patients with combined euthyroid sick syndrome [26]. And Shin et al. also found thyroid hormone replacement therapy attenuated the decline of renal function in chronic kidney disease patients with subclinical hypothyroidism [27]. But for the limited cases, it is hard for our study to conclude whether thyroid hormone replacement would benefit the clinical outcome of patient with combined pMN and thyroid dysfunction or not. Further investigation will be needed.

As a retrospective study, there were some limitations in the current study. First, we did not perform IF/IHC of PLA2R or THSD7A, or other antigens on all the patients’ kidney tissue. Second, the autoantibodies associated with autoimmune thyroiditis were detected in only a few patients. Third, almost half of the patients with thyroid dysfunction were lost to follow-up and the change of thyroid hormones during the follow-up were not detected regularly. And this is a monocentric and single ethnicity study, multiple center studies were needed to validate our results.

In conclusion, thyroid dysfunction is not uncommon in patients with pMN. Patients with combined thyroid dysfunction present with more severe proteinuria, lower level of serum albumin and worse kidney function at the baseline. And thyroid dysfunction might also predict a poor treatment response during follow-up. These findings indicated that thyroid dysfunction may be caused by the massive proteinuria or other immune factors, and could also exacerbate the glomerular damage in return.

## Data Availability

The datasets used and/or analyzed during the current study are available from the corresponding author on reasonable request.
